# Infectious Diseases in General Hospitals

**Published:** 1907-11-16

**Authors:** 


### RESIDENT MEDICAL OFFICERS' DEPARTMENT.

[The Editor accepts no responsibility for any opinions expressed in these columns, which are freely open to all resident medical officers. Discussion and contributions are invited, and, if the latter are accepted, they will be paid for.]

### INFECTIOUS DISEASES IN GENERAL HOSPITALS.

An outbreak of infectious disease may occur at any time in a General Hospital, and the onus of dealing with such generally rests on the resident medical officer. Even with the strictest precautions a case does occasionally arise, and it is only by the exercise of the utmost care by the person responsible that such a catastrophe can be avoided. A short *resumè* of some of the difficulties experienced is instructive. The insidious onset of infectious disease in some children has to be carefully considered. It is well known that acute diseases in children are frequently ushered in by convulsions and high temperature, and for 24 or 48 hours it is often impossible to determine the significance of these symptoms. Again, the parents often unwittingly or deliberately make misleading statements as to the child having had infectious disease or having been exposed to infection. Perhaps a patient may be sent with a note from a medical man saying the case is one suitable for admission, and as a matter of courtesy the case is generally received. Of course no medical practitioner would deliberately send an infectious case for admission, but insidious onsets or atypical forms may mislead even the most careful. Infection may be brought in from the outside by visitors, and for this reason in many children's wards parents and friends are not allowed to visit, and young children are almost invariably excluded as visitors. It must be admitted that it is hard for parents not to be allowed to see their children, but it has been proved by experience that this undoubtedly tends to lessen the risk of infectious diseases occurring in a ward.

In many hospitals there are still isolation blocks attached for the treatment of infectious cases. While it must be granted that under such circumstances an epidemic rarely arises, yet this treatment of infectious cases as part of the work of the hospital is fraught with danger to the patients, for one can never be certain that nurses will strictly carry out all precautions for preventing the spread to the general block.

There are certain infectious diseases which can be treated with perfect safety in the wards of a general hospital. For example, cases of typhoid fever are received in many general hospitals, and if due precautions are taken there is practically no danger of an epidemic occurring in the ward. Again, cases of erysipelas may be treated in a medical ward if everything used by the patient is kept for his sole use, and if the nurse is careful to disinfect her hands after attending to the patient. Even in surgical wards, if a special nurse is allotted to the patient, such cases can be treated; although it must be admitted that such a course is very risky, and is not to be recommended.

In conclusion, it may be laid down that, with the exception of typhoid fever, and, perhaps, of erysipelas in medical wards, cases of infectious disease should not be treated in the wards of a general hospital. The strictest supervision should be exercised by a responsible person to prevent infectious cases being admitted. Any case sent for admission having suspicious symptoms should be isolated for a few days (for in practically all hospitals there are generally one or two side wards where this can be, to a certain extent, carried out). Any case developing suspicious symptoms should be at once isolated, and if infectious disease supervene the case should be sent home or to the hospital for infectious diseases. The ward should then be quarantined, and no fresh cases should be admitted until the full period of quarantine has passed without further cases developing. If further cases should occur the ward must be closed, and all cases discharged as soon as possible, the ward being then thoroughly disinfected in the usual manner. General hospitals should be kept entirely for the treatment of non-infectious cases (except those indicated above), and it would be well for isolation blocks in connection with general hospitals to be abolished. In children's wards the exclusion of parents and friends should be rigidly enforced.

